# Application of mNGS to describe the clinical and microbial characteristics of severe burn a tanker explosion at a tertiary medical center: a retrospective study patients following

**DOI:** 10.1186/s12879-021-06790-5

**Published:** 2021-10-21

**Authors:** Jing Wu, Man Huang

**Affiliations:** Department of Intensive Care Unit, Second Affiliated Hospital, School of Medicine, Zhejiang University, No 1511, Jianghong Rd, Hangzhou, 310009 China

**Keywords:** mNGS, Bloodstream, Severe burn, Fungi, Microbiological

## Abstract

**Background:**

Multiple organ dysfunction syndrome secondary to infection is the leading cause of death in burn patients. Bloodstream infection (BSI) and the prognosis of burn patients are negatively correlated. Metagenomic next-generation sequencing (mNGS) can detect many potential pathogens and may be more valuable for patients with severe burns.

**Methods:**

We retrospectively explored the utility of mNGS in describing the clinical and microbial characteristics of severely burned patients with BSI. We compared mNGS with blood culture.

**Results:**

Fourteen patients (127 blood samples) developed 71 episodes of BSIs with 102 unique causative pathogens. The median total body surface area was 93%. The overall 90-day mortality was 43%. In total, 17 (23.9%) episodes were polymicrobial, and 61 (86.1%) episodes originated from the wound. In total, 62/71 cases (87%) showed positive findings by mNGS, while 42/71 cases (59%) showed positive findings using blood culture. We found that mNGS outperformed culture, especially in terms of fungi (27% vs. 6%, p < 0.0001).

**Conclusions:**

The incidence of BSI and polymicrobial in patients with large-area severe burns is high. mNGS has potential value in the diagnosis of fungal infections and coinfections in such patients. In addition, mNGS may provide unique guidance for antibiotic therapy in complicated BSI.

## Background

Multiple organ dysfunction syndrome secondary to infection is the leading cause of death in burn patients [1, 2], and bloodstream infection (BSI) is the most serious [3]. BSI is an independent factor responsible for mortality in patients with moderate to severe burns [4]. The incidence of infection in patients with a total body surface area (TBSA) over 50% has been reported to be approximately 57% [5], and bloodstream infection and the prognosis of burn patients are negatively correlated [3]. Among patients with large areas of severe burns, due to the small donor area, long hospitalization, immunosuppression, and damaged skin barrier, moist and inactivated tissues are colonized by various organisms, and their proliferation is promoted. The risk of bloodstream infection is extremely high [6]. Prompt and accurate pathogen-targeted therapy is critical for the prognosis of patients. The most commonly used clinical diagnostic methods are still based on the methods introduced by Pasteur in the 1880s. Culture-based technology is time-consuming and has a low positive rate. Metagenomic second-generation sequencing (mNGS) technology is used as an unbiased DNA/RNA detection method [7] that can detect all nucleic acid fragments in an extracted specimen and may have better application value in patients with severe burns in large areas. Accordingly, we performed a retrospective study to explore the utility of mNGS in describing the major microbial trends and characteristics of severely burned patients with BSI.

## Methods

### Patients and microbiological methods

In this single-center retrospective study, we retrospectively identified patients with massive and severe burn injuries due to the “2020.6.13 Wenling Shenhai high-speed oil tanker explosion in China [8]” who were admitted to the intensive care unit (ICU) of the BinJiang campus of the Second Affiliated Hospital of Zhejiang University School of Medicine between June 2020 and September 2020. This study was conducted in accordance with the Declaration of Helsinki (as revised in 2013) and was approved by the Human Research Ethics Committee of the Second Affiliated Hospital of Zhejiang University School of Medicine (ethical number 2020-652) and the institutional review board of the Second Affiliated Hospital of Zhejiang University School of Medicine. Since this study is a retrospective study, the data were anonymous, and the need for informed consent was waived by the Human Research Ethics Committee of the Second Affiliated Hospital of Zhejiang University School of Medicine.

### Inclusion and exclusion criteria

The inclusion criteria were as follows:

1. Patients injured in the “2020.6.13 Wenling Shenhai high-speed oil tanker explosion in China”;
2. Patients treated in our ICU center;
3. Severe burn injury patients with bloodstream infections; and
4. A total burn area ≥ 50% TBSA.

The exclusion criteria were as follows:

1. Patients aged under 18 years;
2. Known pregnancy;
3. Patients treated with antibiotics during the previous 2 weeks; and
4. Patients who died within 72 h of admission.

### Specimen collection and processing

During enrollment, the treating clinicians ordered many blood cultures for all patients. The blood specimens were divided into two equal parts as follows: one part was sent to the microbiological laboratory for a series of standard procedures, and the other part was stored at -20 °C and sent for DNA/RNA extraction and sequencing.

### Blood culture

The patients with persistent clinical manifestations underwent blood culture and laboratory evaluations. The blood culture samples consisted of an aerobic bottle and an anaerobic bottle, and the two sets of samples were collected from two puncture sites. The blood culture bottles were incubated at approximately 37 °C for up to 5 days in the semiautomated continuous monitoring blood culture system BacT/ALERT 3D (BioMérieux, France). Gram staining and subcultures on solid media were performed on positive blood cultures. Microbiological species identification and susceptibility testing were performed in the clinical laboratory by the VITEK 2 system (BioMérieux, France). Multidrug resistance (MDR) was defined as acquired nonsusceptibility to at least one agent in three or more antimicrobial categories [9]. A standard protocol based on the current guidelines [10] was used to manage deep veins. Catheters suspected of contamination were immediately removed and cultured. The catheter placed at the burn site was routinely replaced every 5–7 days and routinely retained for culture. Bacteria were identified at the species level, and the sensitivity was determined according to the Clinical and Laboratory Standards Institute (CLSI) standards [11]. Normal skin flora growing only in a single blood sample was considered contamination and was not included in the analysis.

### mNGS methods

#### PCR-free library preparation and mNGS testing

Whole blood was centrifuged at 1600*g* for 10 min, and the supernatant was centrifuged at 16,000*g* for 10 min to obtain plasma. One milliliter of plasma was pipetted into the cartridge (NGSmasterTM, Matridx Biotechnology Co., Ltd.). A sequencing library was prepared by reverse transcription (for RNA sequencing only), enzymatic fragmentation (except for plasma DNA sequencing since cell-free DNA is intrinsically fragmented), end repair, terminal adenylation, and adaptor ligation. The libraries were quantified by real-time PCR (KAPA) and pooled. Shotgun sequencing was carried out on an Illumina Nextseq. Approximately 20 million 75-bp single-end reads were generated for each library. A bioinformatic analysis was conducted as described in a previous report [12]. Sequences of human origin were filtered (GRCh38.p13), and the remaining reads were aligned to a reference database (NCBI GenBank and in-house curated microbial genomic data) to identify the species and the relative abundance [13]. In each sequencing run, a negative control (plasma from healthy donors) was included.

### Patient management

Once patients were transferred to our center, a multidisciplinary medical team, including nutritionists, rehabilitation therapists, burn and ICU physicians, and infectious disease specialists, was quickly established and held regular meetings daily to discuss treatment and disposal strategies.

All patients received early nutritional support within 24 h after admission and reached full caloric intake guided by indirect calorimetry as soon as possible. In the first 24 h, liquid resuscitation was performed with crystalloids and colloids in a 1:1 ratio according to TBSA, and the pulse index continuous cardiac output and ultrasound were used every 8 h to estimate the central circulatory volume. In addition, all patients underwent tracheotomy and invasive mechanical ventilation, and fiberoptic bronchoscopy was performed for the first time to clear airway secretions. Regarding the surgical timing, escharotomy was performed as soon as possible depending on the patient’s condition. Almost all patients' first operation was completed within 72 h after admission. Only 2 patients’ operations were postponed until 84 h due to extremely unstable vital signs.

### Definitions

Severe burn injury has been defined as an acute burn injury requiring specialized care during hospital admission [14]. Inhalation injury was diagnosed based on previously published criteria [15]. Bloodstream infection (BSI) was defined according to the standards of the American Burn Association [16]. Wound infection was defined as a burn wound culture with > 10^5^ bacteria/g of tissue [16]. Pneumonia, urinary tract infections, CVC infection, enterogenic infection, and bloodstream infection were defined as previously described [16].

The mNGS results were interpreted based on a study conducted by Sen Wang [17] as follows: at least 3 reads were mapped to pathogens whose relative abundance levels surpassed their thresholds as determined by the preliminary sequencing data. Pathogens had the highest absolute abundance in their genus; the pathogens ranked among the top 10 included bacteria, viruses, and parasites, and those ranked among the top 20 included fungi and *Mycobacterium tuberculosis* in relative abundance. After the prior analysis, if the detected pathogens were commonly reported infectious pathogens, they were considered causative agents. If the detected pathogens were uncommonly reported pathogens, the mNGS results were interpreted according to the patient’s clinical features; otherwise, the detected reads were classified as nonpathogenic microbe sequences.

Bloodstream infection (BSI) was defined according to the American Burn Association standard [16]. BSI episodes are defined as different periods of clinical disease associated with positive blood culture/mNGS results. Usually, when an organism is isolated and identified, blood cultures are repeated every 24 h until the next two specimens are negative for the organism, indicating that the incident was resolved.

The onset of infection was defined as the time to the first positive blood culture in each episode. Multimicrobial bloodstream infection refers to an infection with two or more organisms isolated from one or more blood cultures/mNGS runs during the same BSI episode. To establish a clear diagnosis of catheter-related bloodstream infection (CRBSI), the same organism (species and antibacterial sensitivity) must be grown from a blood culture and the catheter tip. Bacteremia has been confirmed in microbiological tests and is considered when determining the status of an infection.

Any one of the following 3 items is considered indicative of bloodstream infection recurrence [18]:

1. New evidence of positive blood cultures/mNGS after clearance during an ongoing antibiotic course;
2. New evidence of positive blood cultures/mNGS in patients with a documented clinical response after completing a course of anti-infection therapy; and
3. Recurrence of circulatory deterioration initially caused by the bloodstream after shock is reversed during proper antibiotic treatment.

### Data collection

The collected data included demographic characteristics, illness severity, isolated pathogen(s), laboratory results, ventilator use, central venous catheter (CVC) use, extracorporeal membrane oxygenation (ECMO) at bacteremia onset, intensive care unit (ICU) hospitalization, and 3-month mortality. The body mass index (BMI) was calculated as kg/m2. The burn severity was evaluated by the Abbreviated Burn Severity Index (ABSI), the presence of a full-thickness burn, and the burned percentage of the total body surface area (TBSA). The revised Baux score was calculated as the sum of age and TBSA. The Acute Physiology and Chronic Health Evaluation II (APACHE II) score was recorded < 24 h after the burn injury in those without bloodstream infection and within 24 h before bloodstream infection onset in those with bloodstream infection [19].

### Statistical analysis

A descriptive analysis of the collected variables was performed, and the included patients exhibited bacteremia. The normally distributed data are expressed as the mean ± standard deviation, and the nonnormally distributed data are expressed as the median and interquartile range. The frequency and corresponding percentage of the qualitative variables were calculated. The proportions were compared using a Chi-squared test or Fisher’s exact test as appropriate. All tests were carried out using bilateral methods, and p < 0.05 was considered significant. p-values < 0.05 were considered significant. The statistical analyses were conducted using SPSS Version 23.0 (IBM Corp., Armonk, NY, USA).

## Results

### Patient demographic and clinical characteristics

In total, 14 burn patients were analyzed in this study. Their demographic characteristics, clinical characteristics, and outcomes are summarized in Table 1. The average age was 67.2 ± 16.8 years (median = 70 years), and 85.7% of the patients were males. Most patients had no comorbidities, and the median Charlson index was 3 (IQR 1.3–4). The median TBSA burn percentage was 93% (IQR 90–98%). The incidence of inhalation injury was 100%. The 90-day total mortality rate was 42.9%.

**Table 1:** Demographics, clinical characteristics, and outcome of severe burn patients with and without bloodstream infections (BSIs)

Variables	n (%)/Mean ± SD/Median (Q1–Q3)
Age in years	67.2 ± 12.8
Gender, Male	12 (85.7)
Body mass index	24.6 ± 3.5
Charlson index	3 (1.3–4)
Total body surface area (TBSA) (%)	93 (90–95)
Second/Third-degree TBSA (%)	93 (90–95)
Inhalation injury	14 (100)
Severity of injury	
Abbreviated burn severity index	15 (13.3–16)
Revised Baux score	176.5 (154.8–187.3)
APACHE II score	24.5 (20.3–26)
SOFA score	10 (5–18)
Invasive procedures	
Ventilation support	14 (100)
Endotracheal tube or tracheostomy	14 (100)
Central venous catheter	14 (100)
Arterial catheter	14 (100)
Hemodialysis	10 (71.4)
Extracorporeal membrane oxygenation	6 (42.9)
Total parental nutrition	4 (28.6)
Laboratory examinations	
White blood cells,/μL	11,000 (8200–15,900)
C-reactive protein, mg/L	141 (107.6–195.7)
Procalcitonin, ng/mL	3.5 (1.6–6.7)
Interleukin-6, pg/ml	362.9 (146.2–842.6)
Clinical characteristics	
Number of operations	6.6 ± 1.8
Number of mNGS	9.4 ± 4.6
Sepsis	14 (100)
Shock	14 (100)
Multiple organ failure	6 (42.9)
Gastrointestinal bleeding	14 (100)
Gastrointestinal dysfunction	7 (50)
Acute respiratory distress syndrome	7 (50)
Ventilation days(d)	42 (28.3–57.5)
Length of ICU stay(d)	66.5 (41.8–86.5)
Length of hospital stay(d)	78 (45.5–96.5)
90 days Mortality	6 (42.9)

### BSI epidemiology

**Fig. 1:**
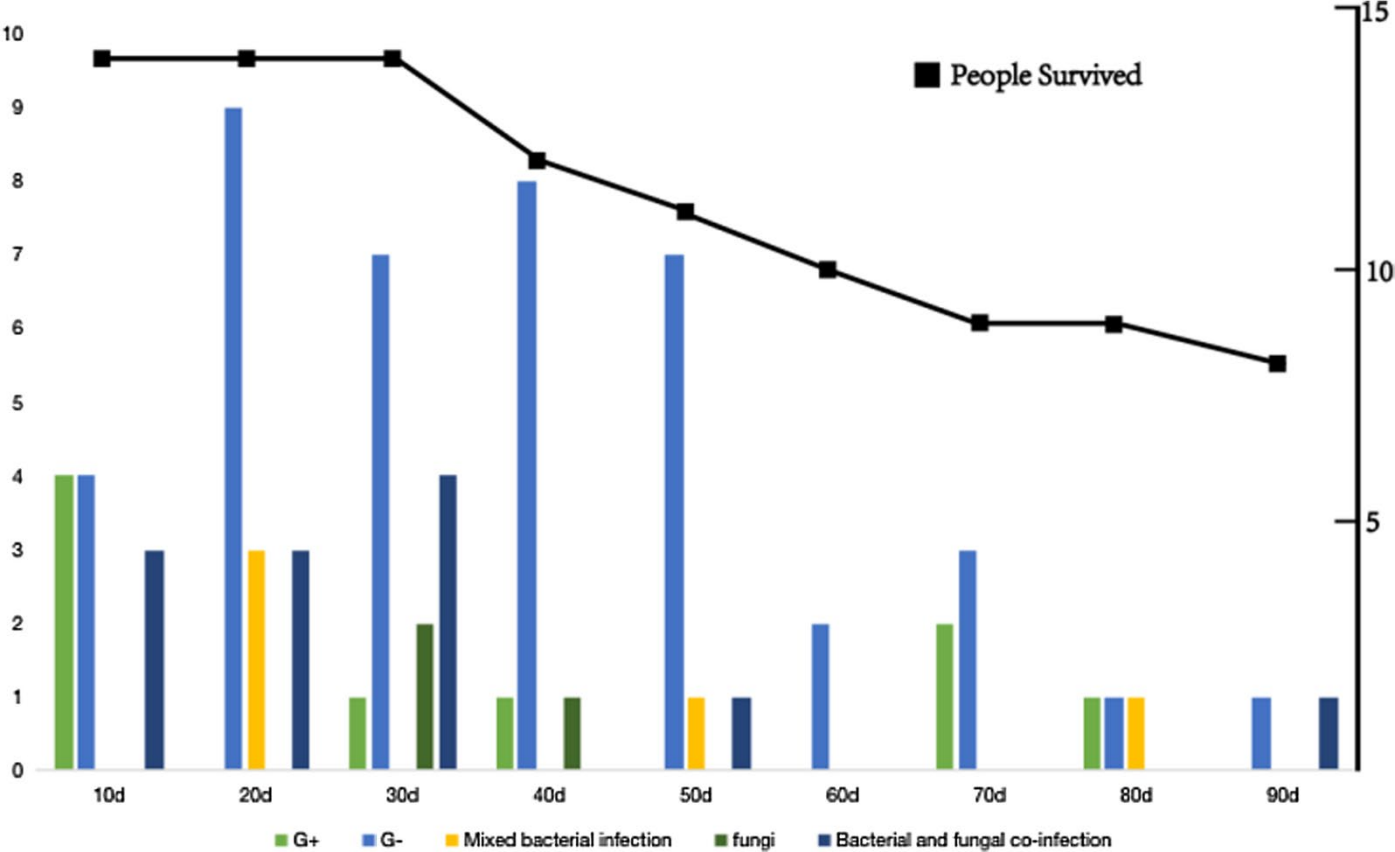
Time distribution of episodes with blood cultures/mNGS, diagnosis of bloodstream infection (71 episodes), and the incidence of G + /G − /Mixed bacterial infection/Fungi/Bacterial and fungal co-infection every 10 days. The black line represents the number of patients surviving at each stage

**Fig. 2:**
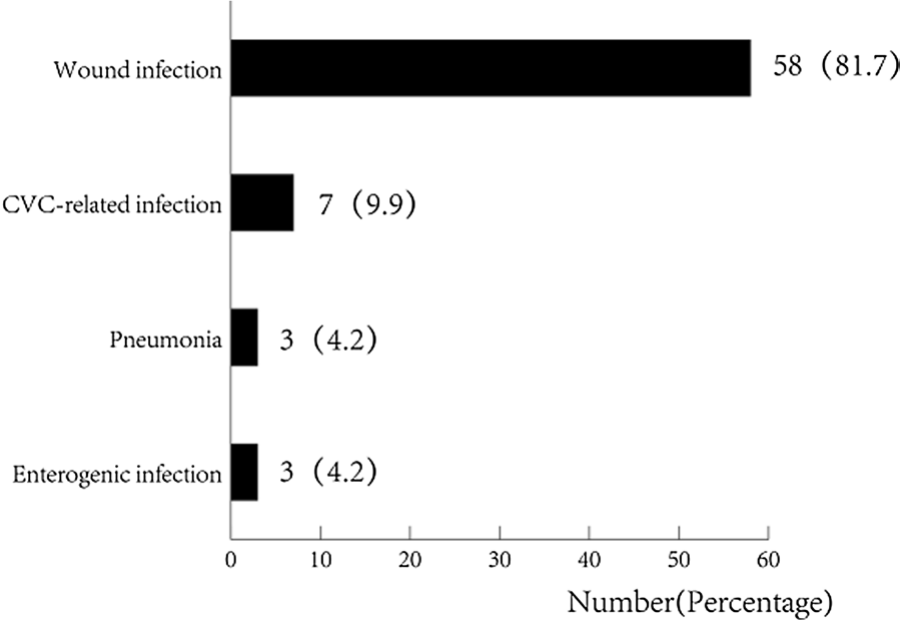
Distribution of the source of infections

During the study period, 127 blood samples were collected from 14 patients, and in total, 71 BSI episodes occurred in 14 patients involving 102 unique pathogenic microbes. Seventeen episodes (23.9%) were polymicrobial infections (including 5 episodes of mixed bacterial infection and 12 episodes of bacterial and fungal coinfection), (Fig. 1). The median time from burn injury to the first BSI was 7 (IQR 3.3–13) days. The incidence of BSI peaked on days 10–20 and gradually decreased (Fig. 1). Among the 71 BSI episodes, 61 cases (86.1%) originated from wounds, 7 cases (9.9%) originated from the central venous catheter (CVC), and 3 cases (4.2%) originated from the lung (Fig. 2).

### Microbiology

Among the 102 pathogens, gram-negative bacteria accounted for 67.6%, gram-positive bacteria accounted for 11.8%, and fungi accounted for 20.6%. The five most common organisms were *Klebsiella pneumoniae, Acinetobacter baumannii, Stenotrophomonas maltophilia, Lichtheimia ramose, Pseudomonas aeruginosa* and *Fusarium* (Table 2). Most isolates cultivated were gram-negative bacteria. However, the proportion of fungal infections was quite high, even surpassing that of gram-positive bacteria. Polymicrobial infections were very common, and most isolates cultured from polymicrobial infections were fungal and gram-negative bacterial infections.

**Table 2:** Microorganisms (n = 102) isolated from 71 episodes of bloodstream infections in patients with severe burn injuries

Causative organisms	Isolates n (%)	Isolates in polymicrobial BSI n (%)
Gram-negative bacteria	69 (67.6)	26 (37.7)
*Klebsiella pneumoniae*	25 (24.5)	5 (20.0)
*Acinetobacter baumannii*	13 (12.7)	4 (30.8)
*Stenotrophomonas maltophilia*	9 (8.8)	8 (88.9)
*Pseudomonas aeruginosa*	7 (6.9)	2 (28.6)
*Burkholderia cepacia*	5 (4.9)	3 (60.0)
*Serratia marcescens*	4 (3.9)	2 (50.0)
*Enterobacter* spp.	3 (2.9)	2 (66.7)
*Morganella morganii*	2 (2.0)	0 (0.0)
Flavobacterium odorum	1 (1.0)	0 (0.0)
Gram-positive bacteria	12 (11.8)	3 (25.0)
* Staphylococcus aureus*	5 (4.9)	1 (20.0)
*Enterococcus* spp.	5 (4.9)	1 (20.0)
Coagulase-negative staphylococci	2 (2.0)	1 (50.0)
Fungi	21 (20.6)	17 (81.0)
Lichtheimia ramosa	8 (7.8)	7 (87.5)
Fusarium	7 (6.9)	5 (71.4)
Aspergillus	3 (2.9)	3 (100.0)
Mucorales	2 (2.0)	2 (100.0)
*Candida glabrata*	1 (1.0)	0 (0.0)

### Comparison of the diagnostic performance of mNGS and culture

According to our results, mNGS identified at least one pathogenic microorganism in 62 (87%) bloodstream infections, and blood culture identified 42 (59%). Compared with the blood cultures, mNGS detected all pathogens (87% vs. 59%, p = 0.0001), fungi (27% vs. 6%, p < 0.0001) and bacteria (85% vs. 52%, p < 0.0001) (Table 3).

**Table 3:** Comparison of diagnostic performance between mNGS and culture (n = 71)

Group	mNGS	Blood culture	P
All pathogen	62	42	0.0001
Bacteria	60	37	< 0.0001
Fungus	19	4	< 0.0001

### Comparative analysis of pathogen types

**Fig. 3:**
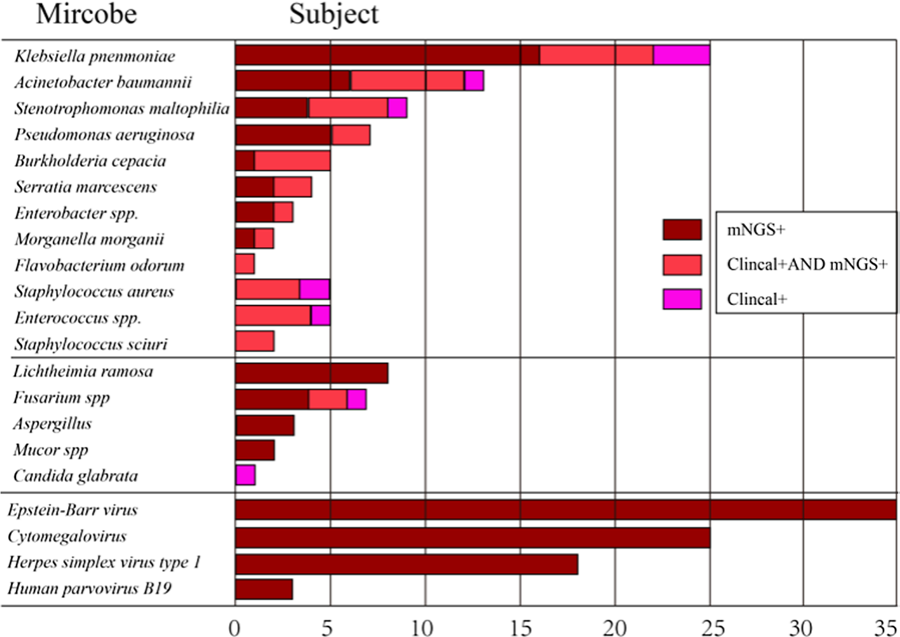
Distribution of the number of subjects in whom each respiratory pathogen was identified using blood cultures versus mNGS. Most microbes detected by blood cultures were also detected by mNGS; *mNGS* metagenomic next-generation sequencing

Among the 102 microorganisms isolated, *K. pneumoniae* (25/24.5%) was the most common pathogen, followed by *A. baumannii* (13/12.7%), *S. maltophilia* (9/8.8%), *Lichtheimia ramosa* (8/7.8), *Fusarium* (7/6.9), and *P. aeruginosa* (7/6.9). All viruses were detected only by mNGS. Among all fungi, higher yields cultured by mNGS were also observed. Interestingly, *Candida glabrata* and *Fusarium* were also occasionally found in the blood cultures (Fig. 3).

**Fig. 4:**
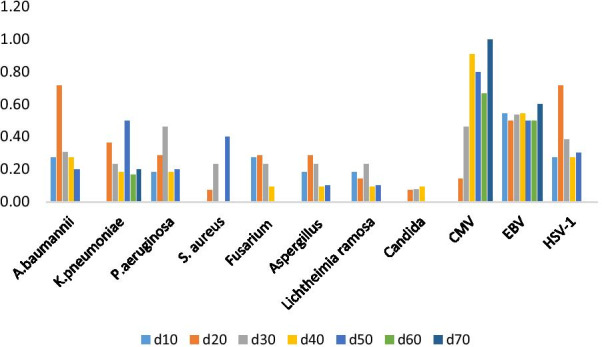
Species distribution of pathogens in different periods after burn injury. The ordinate represents the number of patients examined for this pathogen in different periods/the number of patient samples sent for mNGS in this period

Figure 4 illustrates the trends of microbial isolation in blood by days after admission. Overall, the total number of microbial isolates gradually increased as the number of hospitalization days increased. The microbial isolates markedly increased from days 10 to 20. Fungi were more common in the early stage, CMV and *Staphylococcus aureus* were more common in the late stage, and gram-negative bacteria were more common throughout the entire course of the disease.

### False-negative mNGS results

mNGS missed 10 possible pathogens. Among the 9 “mNGS false-negative” cases (Table 4), the results were inconsistent with the clinical diagnosis. The possible reasons for this inconsistency included severe inflammation (4/9), “weak” positivity (3/9), and culture contamination (1/9), and in 1 case, disruption of the cell wall of *Candida glabrata* was considered difficult. Cases 2–2 and 5–5 were both detected by mNGS after inflammation was slightly controlled.

**Table 4:** False negative of mNGS (n = 9)

Case No	Patient No	Culture	mNGS	Reason
Case 1–3	Case 1	MRSA	Negative	“Weak” positive
Case 2–2	Case 2	CRKP	Negative	Severe inflammatory reaction
Case 2–14	Case 2	CRKP	Negative	Severe inflammatory reaction
Case 3–3	Case 3	Candida glabrata	Negative	Difficult to disrupt cellwall
Case 4–3	Case 4	MRSA	Negative	“Weak” positive
Case 5–5	Case 5	Fusarium	Negative	Severe inflammatory reaction
Case 8–2	Case 8	*Stenotrophomonas maltophilia* Enterococcus faecails	Negative	Culture contaminated
Case 12–2	Case 12	CRKP	Negative	Severe inflammatory reaction
Case 12–5	Case 12	CRAB	Negative	“Weak” positive

*MRSA* methicillin-resistant *Staphylococcus aureus*, *CRKP* carbapenem-resistant Klebsiella pnermoniae, *CRAB* carbapenem-resistant *Acinetobacter baumannii*

### Significance of continuous mNGS inspection

In our study, in total, 14 patients developed 71 episodes of bloodstream infections, and 14 episodes occurred in Case 2, all of which were *carbapenem-resistant Klebsiella pneumoniae* (CRKP) bloodstream infections. The patient’s clinical symptoms suggested the presence of infection; the blood cultures only detected 3 episodes, and the remaining 11 episodes were detected by mNGS.

### Turnaround time

In the mNGS group, the turnaround time ranged from 20 to 40 h, with an average time of 24.1 ± 2.49 h, which was significantly shorter than the turnaround time of 72 h or longer in the culture group (77.1 ± 13.3 h) (p < 0.0001).

### Impact on antibiotic treatment

The records of antibiotic treatment during hospitalization for all 71 BSI episodes that occurred in 14 patients were retrieved. Based on the microbiologic mNGS testing results, the number or spectrum of antimicrobial agents was de-escalated in 6 (8.5%) patients, escalated in 29 (40.8%) patients, and unchanged in 36 (50.7%) patients (Table 5). Most escalation events were related to the use of antimicrobial agents against specific pathogens, especially antifungal drugs.

**Table 5:** Estimated potential impact of mNGS testing on application of antimicrobial agents in patients with severe burn patients (n = 71)

Modifications	Antimicrobial agents	*N* (%)
De-escalation		6 (8.5)
Antifungal	Remove VCZ	2
	Reduce AmB to CAS	1
Against Gram-negative bacilli	Reduce MEM to SCF	2
	Reduce SCF to CAZ	1
Escalation		29 (40.8)
Antifungal	Add VCZ	5
	Add AmB	4
	Add CAS	2
Against Gram-negative bacilli	Add polymyxin B	3
	Add TGC	4
	Add ceftazidime and avibactam	2
	Add AmK	1
	Add MEM	2
	Add TGC + MEM	1
Against Gram-postive bacilli	Add VAN	2
	Add LZD	3
No change		36 (50.7)

*VCZ* voriconazole, *AmB* amphotericin B, *CAS* caspofungin, *MEM* meropenem, *SCF* sulbactam and cefoperazone, *CAZ* ceftazidime, *TGC* tigecycline, *AmK* amikacin, *VAN* vancomycin, *LZD* linezolid

## Discussion

Although many studies have evaluated bloodstream infections in severe burn patients, the results of these studies are based on blood culture results. To the best of our knowledge, this study is the first to describe the utility of mNGS in severe burn patients with bloodstream infection. First, a large area of severe burns is associated with a high risk of infection and high mortality [3, 5], and antibiotic use in such patients is very powerful [19]. In this situation, the positive rate of blood culture is considerably reduced. In addition, the incidence of fungal infections in elderly severe burn patients is very high [20], and blood cultures are insufficient for fungal detection due to the sensitivity deficiency and slow multiplication [21, 22]. As an unbiased DNA/RNA detection method, mNGS is widely used in clinical practice but is rarely used in burn patients [23]. Therefore, we explored the application of mNGS in severely burned patients with BSI. We found that the incidence of bloodstream infections in severe burn patients was very high, and the diagnostic value of mNGS was better than that of blood culture in this population, especially for diagnosing fungemia.

Our results were found in a series of 14 burn patients who experienced 71 documented BSIs, which is higher than the figure reported in previous decades [24, 25]. First, the populations included are different. We included older patients with severe and extensive burns. Most previous studies did not focus on large-area burns in an elderly population, which represent the main risk factor for bloodstream infection in burn patients [26, 27]. Then, the positive rate of blood culture was markedly reduced due to the use of strong antibiotics, and mNGS can avoid these issues to a certain extent.

The 90-day mortality rate of the 14 elderly patients with severe burns in our study was as high as 40%, which is higher than that in other studies [24, 25]. In addition to age and extensive burns, another reason that cannot be ignored is the higher proportion of earlier fungal infections in these patients, especially *Lichtheimia ramosa* and *Fusarium* infections, which may be related to most of our patients working on farmland when the explosion occurred. *Lichtheimia ramose* and *Fusarium* can widely exist in soil and water [28]. In addition to environmental reasons, fungal infection may be related to an advanced age and a large TBSA. The average age of our patients was 67 years, and the average TBSA was 75%. In addition, due to the patients’ unstable vital signs, the first operation was completed within 72–84 h after admission, which may also be one of the factors in early fungal infection. In previous studies, bloodstream fungal infections were mostly caused by *Candida*, and *Candida* infections are known to mostly originate from central venous catheters. Due to our series of infection control measures, candidemia was substantially reduced. We confirmed that a patient had an *Aspergillus* bloodstream infection by the presence of large mold plaques in the wound accompanied by unstable circulation and pathological support. mNGS is an unbiased detection method [23] that has been confirmed in previous studies to have unique advantages in the detection of fungi, viruses, and atypical pathogens [29, 30]. Our research also shows its absolute advantage in fungal detection in severe burn patients. Notably, when inflammation is extremely severe and the personnel sequence is highly variable, mNGS may also produce false-negative results.

In our study, the detection of herpes simplex virus, especially human herpes virus-1 (HSV-1), Epstein-Barr virus (EBV), and cytomegalovirus (CMV), was common. The detection rate of EBV can reach 49% (35/71) and that of CMV can be 35% (25/71), which is similar to other studies [31, 32]. Interestingly, EBV remained uniform in 50% of the cases in the entire course, but CMV showed a gradually increasing trend, which is considered related to the suppression of the immune function of these patients. We did not use antiviral drugs in any patients and found no evidence of significant viral infections. We consider reactivation of a virus indicative of an immunosuppressed state. The relationship between the immune status and cytomegalovirus activation requires further investigation.

In our research, we found that more than 80% of the infections were from wounds, far exceeding the data in previous reports. In the previous literature, the rate of wound infection was approximately 50–60% [24, 25], and the incidence rates of catheter-related infections and pneumonia were extremely low, which may be related to the following reasons. First, the burn area in most of our patients was greater than 90%. As the burn area increases, the risk of wound infection increases [26, 27]. Second, our center adopted strict infection control measures, and CVCs did not pass through the wounds and were replaced within 5 days. If any signs of infection were noted, the catheter was immediately replaced. In addition, we adopted a shallow sedation strategy, maintained RASS scores of 0–1 points, and implemented bed turning and transposition every 4 h, which substantially reduced the occurrence of ventilator-related pneumonia, providing further insight into lung management in other nonburn patients. In addition, we observed 3 episodes of intestinal infection in 1 patient with extremely high intra-abdominal pressure, but this finding was actually a typical manifestation of wound sepsis; thus, this case was strictly classified as a wound infection.

In our study, many patients underwent mNGS several times. On average, each patient underwent mNGS 9.4 times, and mNGS was performed 22 times in Case 2. In Case 2, the patient experienced 14 episodes of bloodstream infection in 82 days. Among these 14 episodes, only 3 blood cultures identified the pathogen, and the remaining 11 episodes were detected by mNGS. In Case 7, blood culture and mNGS both showed infection with *Morgan morganii*. However, fever persisted even after using imipenem according to drug sensitivity and two consecutive negative blood cultures. Considering that the patient still had an infection, mNGS was performed again, which indicated that the *Morganella morganii* infection remained.

Our study is not without limitations. First, this study was a retrospective study with some inherent defects in the study design, such as inevitable selection bias and recall bias. Second, the sample size in this study was small. Third, because we conducted the study in severe burn patients from a tanker disaster, the results should be interpreted with caution with respect to generalization to other critically ill patients. Finally, the study was conducted in a single facility, and multicenter studies with larger samples are needed to externally validate our results.

## Conclusion

The incidence of bloodstream infections in patients with large-area severe burns is high, and the rate of polymicrobial infections is high. mNGS has potential value in the diagnosis of fungal infections and coinfections in such patients. In addition, mNGS may provide unique guidance for antibiotic courses in complicated bloodstream infections.

## Data Availability

All data generated or analyzed during this study are included in this published article.

## Abbreviations

mNGS: Metagenomic next-generation sequencing
TBSA: Total body surface area
ICU: Intensive care unit
MDR: Multidrug resistance
CLSI: Clinical and Laboratory Standards Institute
BSI: Bloodstream infection
CRBSI: Catheter-related bloodstream infection
CVC: Central venous catheter
ECMO: Extracorporeal membrane oxygenation
BMI: Body mass index
ABSI: Abbreviated burn severity index
APACHE II: Acute physiology and chronic health evaluation II
CRKP: Carbapenem-resistant *Klebsiella pneumoniae*
MRSA: Methicillin-resistant *Staphylococcus aureus*
CRAB: Carbapenem-resistant *Acinetobacter baumannii*
HSV-1: Human herpes virus-1
EBV: Epstein-Barr virus
CMV: Cytomegalovirus
VCZ: Voriconazole
AmB: Amphotericin B
CAS: Caspofungin
MEM: Meropenem
SCF: Sulbactam and cefoperazone
CAZ: Ceftazidime
TGC: Tigecycline
AmK: Amikacin
VAN: Vancomycin
LZD: Linezolid
